# Supplementary material for: Benchmarking Algorithms for Gene Set Scoring of Single-cell ATAC-seq Data
Source: Genomics Proteomics Bioinformatics. 2024 Feb 9;22(2):qzae014. doi: 10.1093/gpbjnl/qzae014 (PMC11423854; doi:10.1093/gpbjnl/qzae014)
Supplement: qzae014_Supplementary_Data [file qzae014_supplementary_data.zip › Figure S1.pptx]

## Slide 1
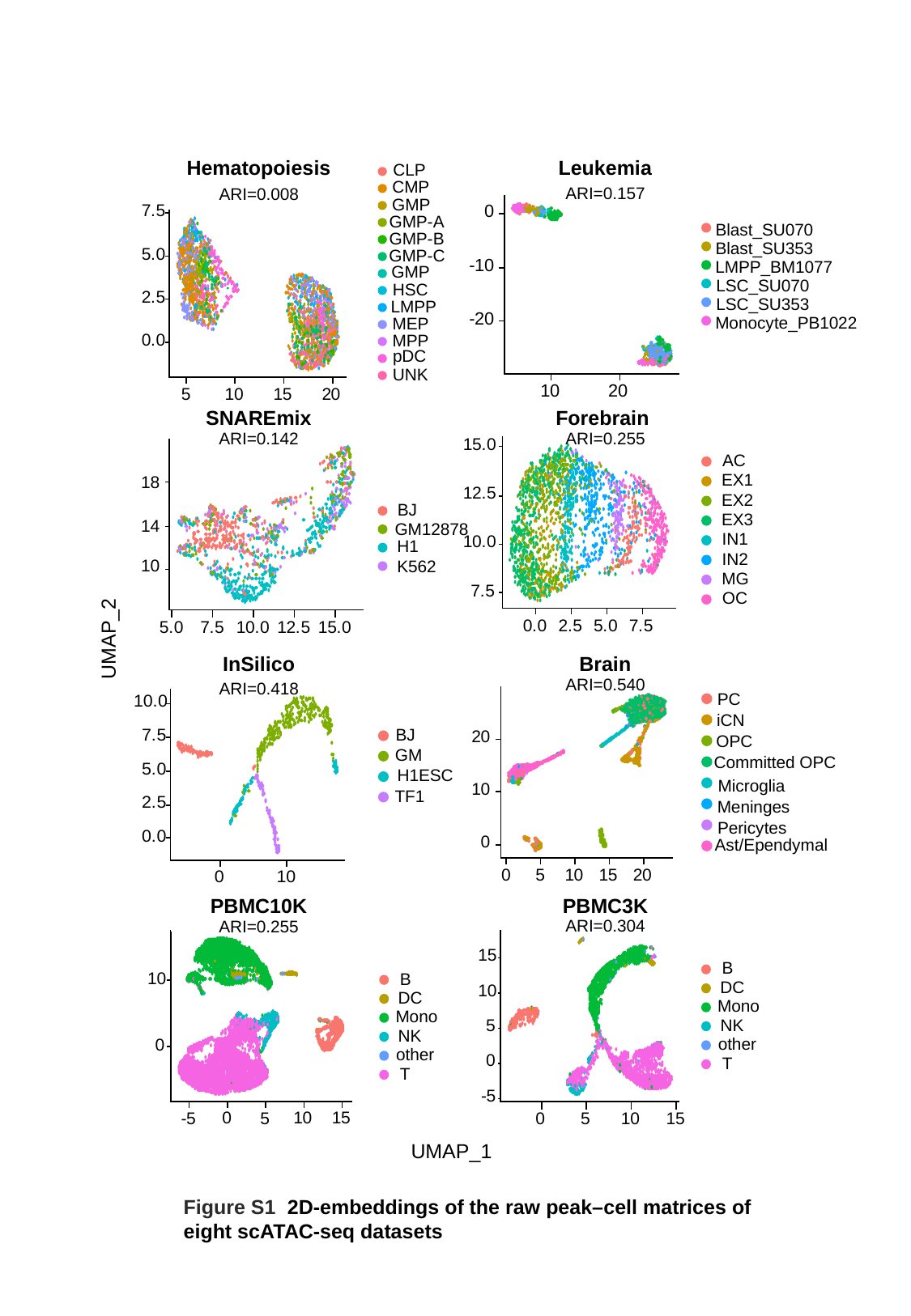

Hematopoiesis
Leukemia
CLP
CMP
GMP
GMP-A
GMP-B
GMP-C
GMP
HSC
LMPP
MEP
MPP
pDC
UNK
ARI=0.157
ARI=0.008
Blast_SU070
Blast_SU353
LMPP_BM1077
LSC_SU070
LSC_SU353
Monocyte_PB1022
SNAREmix
Forebrain
ARI=0.142
ARI=0.255
AC
EX1
EX2
EX3
IN1
IN2
MG
OC
BJ
GM12878
H1
K562
UMAP_2
InSilico
Brain
ARI=0.540
ARI=0.418
PC
iCN
OPC
Committed OPC
Microglia
Meninges
Pericytes
Ast/Ependymal
BJ
GM
H1ESC
TF1
PBMC10K
PBMC3K
ARI=0.304
ARI=0.255
B
DC
Mono
NK
other
T
B
DC
Mono
NK
other
T
UMAP_1
Figure S1 2D-embeddings of the raw peak–cell matrices of eight scATAC-seq datasets
