# Supplementary material for: Benchmarking Algorithms for Gene Set Scoring of Single-cell ATAC-seq Data
Source: Genomics Proteomics Bioinformatics. 2024 Feb 9;22(2):qzae014. doi: 10.1093/gpbjnl/qzae014 (PMC11423854; doi:10.1093/gpbjnl/qzae014)
Supplement: qzae014_Supplementary_Data [file qzae014_supplementary_data.zip › Supplementary material captions.docx]

**Supplementary materials**

**Figure S1 2D-embeddings of the raw peak–cell matrices of eight scATAC-seq datasets**

**Table S1 Size of gene set collections used in this study**

**Table S2 Detailed information of scATAC-seq and scRNA-seq datasets used in this study**

**Table S3 Human marker gene sets collected from the CellMarker database**
